# Deep brain stimulation modulates nonsense-mediated RNA decay in Parkinson’s patients leukocytes

**DOI:** 10.1186/1471-2164-14-478

**Published:** 2013-07-16

**Authors:** Lilach Soreq, Hagai Bergman, Zvi Israel, Hermona Soreq

**Affiliations:** 1 Department of Medical Neurobiology, Institute of Medical Research Israel-Canada, Hadassah Medical School, The Hebrew University, Jerusalem, Israel; 2 The Edmond and Lily Safra Center for Brain Sciences, The Hebrew University, Jerusalem 91904, Israel; 3 Center for Functional & Restorative Neurosurgery, Department of Neurosurgery, Hadassah University Hospital, Jerusalem, Israel; 4 The Department of Biological Chemistry, The Life Sciences Institute, The Hebrew University of Jerusalem, Jerusalem, Israel

**Keywords:** Alternative splicing, Deep brain stimulation, Exon microarrays, Leukocytes, Nonsense-Mediated decay, Parkinson’s disease

## Abstract

**Background:**

Nonsense-Mediated decay (NMD) selectively degrades mRNA transcripts that carry premature stop codons. NMD is often triggered by alternative splicing (AS) modifications introducing such codons. NMD plays an important regulatory role in brain neurons, but the *in vivo* dynamics of AS and NMD changes in neurological diseases and under treatment were scarcely explored.

**Results:**

Here, we report exon arrays analysis of leukocyte mRNA AS events prior to and following Deep Brain Stimulation (DBS) neurosurgery, which efficiently improves the motor symptoms of Parkinson’s disease (PD), the leading movement disorder, and is increasingly applied to treat other diseases. We also analyzed publicly available exon array dataset of whole blood cells from mixed early and advanced PD patients. Our in-house exon array dataset of leukocyte transcripts was derived from advanced PD patients’ pre- and post-DBS stimulation and matched healthy control volunteers. The mixed cohort exhibited 146 AS changes in 136 transcripts compared to controls, including 9 NMD protein-level assessed events. In comparison, PD patients from our advanced cohort differed from healthy controls by 319 AS events in 280 transcripts, assessed as inducing 27 protein-level NMD events. DBS stimulation induced 254 AS events in 229 genes as compared to the pre-DBS state including 44 NMD inductions. A short, one hour electrical stimulus cessation caused 234 AS changes in 125 genes compared to ON-stimulus state, 22 of these were assessed for NMD. Functional analysis highlighted disease-induced DNA damage and inflammatory control and its reversal under ON and OFF stimulus as well as alternative splicing in all the tested states.

**Conclusions:**

The study findings indicate a potential role for NMD both in PD and following electrical brain stimulation. Furthermore, our current observations entail future implications for developing therapies for PD, and for interfering with the impaired molecular mechanisms that underlie PD and other neurodegenerative and neurological disorders, as well as DBS-treatable conditions in general.

## Background

NMD is an eukaryotic mRNA surveillance process that detects, and selectively degrades, mRNA transcripts which harbour premature termination codons (PTC; [1]), increasing the fidelity of gene expression by degrading aberrant mRNAs that, if translated, would produce truncated proteins [2]. The complex NMD mechanism is required to distinguish a normal stop codon from a premature one, with both splicing and translation crucial for this discrimination in mammals [3].

**Figure 1:**
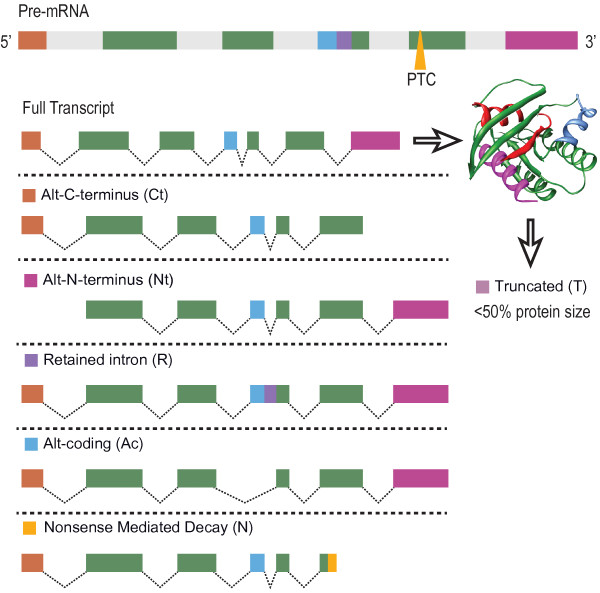
**Types of protein-level functional implications of interrogated AS events.** The AS events detected in both whole blood and leukocyte exon array data sets of PD patients and healthy control (HC) volunteers, and pre- and post- DBS treatment, included alternative N and C termini, retained introns, alternative coding of internal gene regions, NMD and truncation (>50% reduction in protein product size). Top: a schematic structure of a gene. Grey: introns, color coded regions: exons. Direction: from 5’ to 3’ UTR. PTC: Pre-Termination codon. Below it, the structure of a full transcript is given, with a theoretical protein product structure.

The pre-mRNA splicing machinery recognizes a termination codon as premature when it is located more than about 50 nucleotides upstream of the final intron position. Therefore, NMD can result from somatic DNA rearrangements, the presence of upstream open reading frames, the use of alternative open reading frames or the presence of UGA codons [4-7]. More than a third of reliably inferred Alternative Splicing (AS) events in humans result in PTC-containing mRNA isoforms [8], and alternative usage of promoter and polyadenylation sites often places a translation termination codon in a premature context and thereby triggers NMD [9,10]. Thus, many alternative mRNA isoforms carry PTCs that render them as NMD targets and exhibit AS-induced NMD in humans [11]. Examples include transcripts carrying an exon-exon junction downstream of the stop codon, which induces NMD by recruiting both splicing and translation termination factors. In some cases, AS can truncate the normal protein product by causing a frame shift [12]. This may simply remove a protein functional domain, or trigger NMD if the new translation stop site is located too far upstream from the original stop site [13]. Thus, AS contributes to targeting mRNAs for NMD [14]. Also, alternative polyadenylation sites may trigger NMD by generating a long 3′-untranslated region (3′-UTR). Notably, it is estimated that about 95% of the human genes undergo AS changes which create numerous transcript variants from a single gene [15]. This may induce alternative C-or N-terminal sequences in the produced protein, or lead to retained introns, alternative coding of internal gene regions (i.e. exon inclusion or exclusion) and may trigger NMD changes (Figure 1). Correspondingly, mal-functioning NMD leads to mis-regulation of a significant fraction of mRNAs [16]. For example, the core small nuclear ribonucleoprotein (snRNP) SmB/B′ self-regulates its expression by promoting the inclusion of a highly conserved alternative exon in its own pre-mRNA that targets the spliced transcript for NMD [17]. While NMD factors are essential in early embryonic development [18], the key physiological roles of NMD are largely unknown.

That NMD functions as a quality control mechanism tentatively suggested involvement of this particular RNA surveillance process in both the initiation and progression of neurodegenerative diseases and in treatment-induced modifications of disease symptoms. Specifically, we considered the rapid changes occurring following Deep Brain Stimulation (DBS) treatment, a functional stereotactic neurosurgery that is most commonly applied to ameliorate Parkinson’s disease (PD) motor symptoms through targeting of the Sub-Thalamic Nucleus (STN) brain region [19]. Following treatment, the normalized neuronal activity then conveys rapid signals to peripheral tissues, such that DBS improves the four cardinal PD motor symptoms: tremor, rigidity, bradykinesia (“slow movement”), akinesia (“lack of movements”) [20] and postural instability [20,21]. Overall, DBS has been shown to be particularly beneficial for PD patients with severe motor complications [22] and it has the unique advantage of reversibility and adjustability over time [23]. In recent years, the use of DBS has rapidly expanded both for other movement disorders and for conditions such as treatment-refractory depression, epilepsy and psychiatric diseases, but the underlying molecular mechanisms governing its effects remained unknown.

Although PD is a central nervous system disease, the current estimation is that it initiates up to decades prior to the appearance of the motor symptoms [24], including autonomic nervous system involvement. The spatio-temporal distribution pattern of PD-related inclusions includes lesions in olfactory structures, projection neurons and the enretic nervous system [25]. Moreover, PD is also characterized by non-motor manifestations [26]. In recent years, evidence for the presence of blood RNA biomarkers for PD was accumulated from numerous, independent studies [27-29]. Moreover, although PD symptoms reflect preferential neuronal death, DNA, RNA and biochemical traits of the disease are detectable in blood cells [30-32]. We [33] and others [34] have employed exon arrays to identify genome-scale transcriptional [35] and AS aberrations [33] in blood cells and leukocytes from PD patients before and after DBS treatment (i.e. ON stimulation) as compared with healthy control (HC) volunteers and with patients with disconnected electrical stimulation which lasted only one hour. The short electrical stimulation cessation rapidly re-induces the disease motor symptoms, suggesting involvement of fast brain-to-body communications. Most recently, we have managed to classify brain transcriptomes of an external cohort of PD patients from control samples based on AS signatures detected in our cohort of PD patients’ blood leukocytes and matched HC [36]. Leukocytes, in contrast to whole blood cells, can also report or respond to brain infarction, for example in stroke [37].

In our current study, we zoomed in to profile NMD events in leukocyte transcripts from PD patients before and after DBS neurosurgery and under disconnected electrical current, aiming to challenge the hypothesis that NMD may be actively involved in the impaired brain-to-body signalling processes that accompany PD and in the correction of such impairments under DBS which mitigates PD symptoms. We predicted NMD profile changes in PD leukocytes compared to healthy volunteers and in post-DBS compared to pre-DBS preparations, and surmised that disconnecting the electrical stimulus would again alter the NMD profile. To challenge these predictions, we integrated a web-available exon array data set of human PD blood samples [34] with our own PD blood leukocyte exon array datasets of patients pre- and post-DBS and characterized the corresponding NMD events. Here, we provide evidence for NMD profile changes that are compatible with a regulatory role of NMD in both PD and under DBS ON Stimulation as well as following an hour stimulation cessation (i.e. OFF stimulation). To our knowledge, this is the first evidence that electrical stimulation in the brain induces NMD changes in disease-relevant blood cell transcripts, and that these changes are rapidly susceptible for reversal.

## Results

### AS events in blood mRNA from mixed early and advanced PD patients induce a small number of NMD events

**Figure 2:**
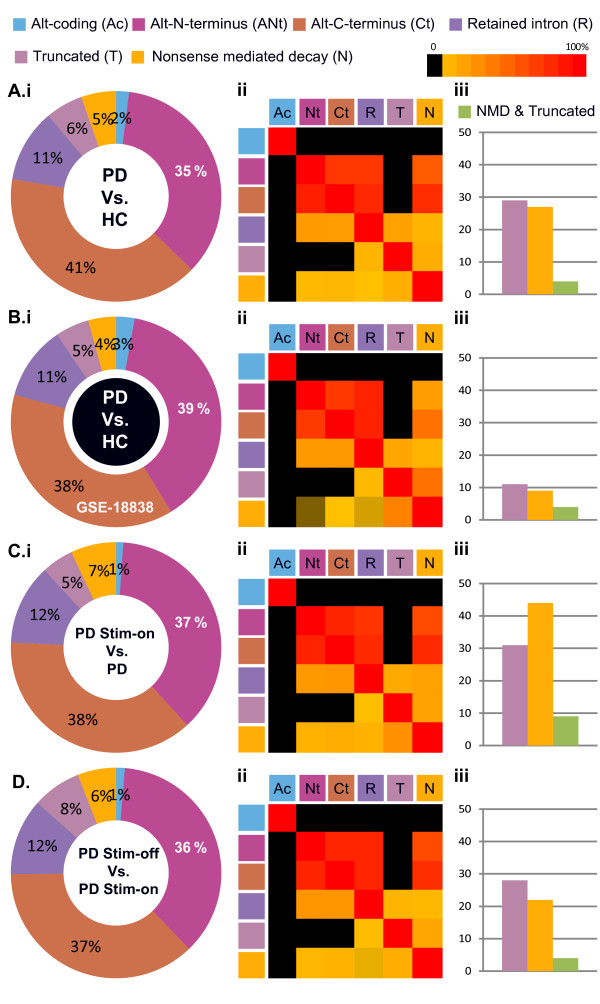
**Post-DBS increases in AS-induced NMD events. A**-**D**: Shown are total numbers of AS events (i), relative frequency of AS events in exons (ii), and overlapping ratio between functional predictions of the changed exons (black denotes no overlap) (iii) that were detected by exon. Exon level analysis of exon array data from (**A**) whole blood from mixed early-advanced PD patients external cohort and HC volunteers (**B**) advanced PD patients as compared with control volunteers (**C**) advanced patients post-DBS on stimulation (PD Stim-ON) as compared with pre-DBS and (**D**) post-DBS OFF stimulus as compared with one hour earlier ON- stimulus. (**E**): Total numbers of NMD and truncation AS events detected in each of the tested conditions. Purple: truncation events, orange: NMD, green: both. The NMD events are a subset of the total number of detected AS events that were observed in all of the interrogated microarray probe-sets.

To characterize alternative splicing changes in exon array datasets from PD patients, we first studied our in-house exon array leukocyte dataset (of overall 27 samples) by measuring the exon level splicing index and assessing functional protein-level implications of these measures in our data-set, as well as in an additional independent exon array dataset of whole mRNA extracted from blood samples of mixed early and advanced PD patients and healthy control volunteers (overall 28 samples) [34]. Exon level splicing-index (SI) analysis of the mixed published cohort interrogated 27,305 transcripts, yielded 146 AS events (defined as presenting either SI or MiDAS p-value < 0.05 and at least 2-fold change) that occurred in 136 genes (Figure 2Ai and Additional file 1: Table S1). Overall, 217 functional changes were predicted in the corresponding protein products for the detected AS transcripts, the most abundant of which were altered N- and C- termini (together comprising 77% of all changes, Figure 2Aii). Of these, a total of 9 AS changes were predicted to induce NMD protein-level changes in patients compared to controls (Additional file 1: Table S1). On average, 67% of the events were associated with more than one functional event, and alternative N- terminus showed the largest overlap with other functional predictions (Figure 2Aiii). The detected genes included the kinesin family member 1 binding protein KIAA1279, previously identified as related to cortical brain malformations [38]. One of the mixed cohort detected genes, RPL10 also showed general gene level expression change detected previously by transcript-level analysis of our advanced PD patient’s cohort exon array data [35]. Functional enrichment analysis of the AS genes detected as changed in the mixed cohort identified ATP binding, alternative splicing and transition metal ion binding (Table 1 and Additional file 2: Table S2).

**Table 1:** High enrichment for alternative splicing pre- and post-DBS on and off stimulation

**Type**	**Category**	**Term**	**Count**	**%**	**p-value**
**PD**	SP_PIR_KEYWORDS	cytoplasm	72	2.875	**0.000**
	GOTERM_CC_FAT	GO:0043232 ~ intracellular non-membrane-bounded organelle	57	2.205	**0.000**
	SP_PIR_KEYWORDS	phosphoprotein	128	4.952	**0.000**
	GOTERM_CC_FAT	GO:0005819 ~ spindle	11	0.426	**0.000**
	SP_PIR_KEYWORDS	alternative splicing	129	4.990	**0.000**
	UP_SEQ_FEATURE	splice variant	129	4.990	**0.000**
	GOTERM_CC_FAT	GO:0015630 ~ microtubule cytoskeleton	20	0.774	**0.000**
	GOTERM_CC_FAT	GO:0005856 ~ cytoskeleton	35	1.354	**0.000**
	GOTERM_CC_FAT	GO:0044430 ~ cytoskeletal part	26	1.006	**0.000**
	GOTERM_CC_FAT	GO:0031974 ~ membrane-enclosed lumen	40	1.547	**0.001**
	GOTERM_BP_FAT	GO:0033554 ~ cellular response to stress	19	0.735	**0.001**
	SP_PIR_KEYWORDS	cytoskeleton	20	0.774	**0.001**
	SP_PIR_KEYWORDS	atp-binding	31	1.199	**0.002**
	GOTERM_MF_FAT	GO:0004674 ~ protein serine/threonine kinase activity	15	0.580	**0.003**
	GOTERM_BP_FAT	GO:0006259 ~ DNA metabolic process	16	0.619	**0.003**
	GOTERM_CC_FAT	GO:0070013 ~ intracellular organelle lumen	36	1.393	**0.003**
	GOTERM_BP_FAT	GO:0032092 ~ positive regulation of protein binding	3	0.116	**0.004**
	GOTERM_CC_FAT	GO:0005815 ~ microtubule organizing center	10	0.387	**0.004**
	GOTERM_MF_FAT	GO:0000166 ~ nucleotide binding	46	1.779	**0.005**
	GOTERM_CC_FAT	GO: 0005694 ~ chromosome	14	0.542	**0.005**
	GOTERM_CC_FAT	GO:0043233 ~ organelle lumen	36	1.393	**0.005**
	SP_PIR_KEYWORDS	acetylation	50	1.934	**0.005**
	GOTERM_CC_FAT	GO:0070161 ~ anchoring junction	8	0.309	**0.006**
	GOTERM_MF_FAT	GO:0005524 ~ ATP binding	33	1.277	**0.006**
	SP_PIR_KEYWORDS	nucleotide-binding	35	1.354	**0.006**
	GOTERM_CC_FAT	GO:0005813 ~ centrosome	9	0.348	**0.007**
	GOTERM_MF_FAT	GO:0032559 ~ adenyl ribonucleotide binding	33	1.277	**0.008**
	SP_PIR_KEYWORDS	transferase	30	1.161	**0.008**
	GOTERM_BP_FAT	GO:0030099 ~ myeloid cell differentiation	6	2.232	**0.008**
	GOTERM_BP_FAT	GO:0009057 ~ macromolecule catabolic process	20	0.774	**0.008**
	GOTERM_CC_FAT	GO:0019898 ~ extrinsic to membrane	14	0.542	**0.009**
	GOTERM_MF_FAT	GO:0030554 ~ adenyl nucleotide binding	34	1.315	**0.009**
**DBS**	UP_SEQ_FEATURE	FEATURE splice variant	115	53.488	**0.000**
	SP_PIR_KEYWORDS	phosphoprotein	112	52.093	**0.000**
	SP_PIR_KEYWORDS	alternative splicing	114	53.023	**0.000**
	UP_SEQ_FEATURE	domain:EH 1	3	1.395	**0.002**
	UP_SEQ_FEATURE	domain:EH 2	3	1.395	**0.002**
	GOTERM_CC_FAT	GO:0005829 ~ cytosol	25	11.628	**0.007**
**OFF**	SP_PIR_KEYWORDS	alternative splicing	63	55.263	**0.000**
	UP_SEQ_FEATURE	splice variant	61	53.509	**0.001**

The functional terms as well as sequence feature and domains detected as the highest enriched in spliced transcripts of leukocyte RNA extracted from blood samples of advanced PD patients’ pre-DBS and post-DBS ON- and following one hour OFF- stimulus (p < 0.01) are shown. Red: the repeatedly appearing term *alternative splicing.* Given are: the compared state (PD: patients pre-DBS compared with control volunteers, DBS: PD patients post-DBS on electrical stimulation as compared with pre-DBS state, OFF: PD patients post-DBS following one hour of electrical stimulation cessation. Category: the type of term, term: the name of the term (and Gene Ontology – GO- ID for GO terms), count: the number of spliced transcripts that are associated with the corresponding term, %: the percent of the spliced transcripts in the term, p-value: statistical significance.

### AS-triggered NMD events in advanced PD leukocytes as compared with healthy controls

Our dataset differed from the published one in two principal points: both the clinical characteristics of recruited patients (advanced stage as compared to mixed composition) and the population of blood cells from which RNA was extracted (purified leukocytes as compared to the total variety of blood cells). Overall, 319 AS events were detected in the advanced patients compared to control volunteers (Figure 2Bi). These included 175 inclusion and 144 exclusion events, which totally occurred in 280 genes (Additional file 3: Table S3). These spanned the innate immunity gene FBXO9 [39] and LRRC8C, which we identified as a PD leukocyte signature gene, based on its transcript level changes [35]. Five spliced transcripts were identified both in this mixed cohort and in our advanced PD patients’ blood leukocytes: BTNL8, RAD17, ARL17B, UTY and DROSHA (suggesting possible relevance of microRNA regulation of the detected changes). Of the detected AS changes, 284 were predicted to cause functional change at the protein level (i.e. alternative N- or C- termini, NMD, truncation, change of alternative coding regions and intron retention). The majority of those were alternative C-termini (41%) and N-termini (35%) (Figure 2Bii and Additional file 3: Table S3). On average, 63% of the identified AS events predictably induce more than one functional change (Figure 2Biii), with 28 predicted to induce NMD (Figure 2Bii, E). Notably, in spite of the more heterogeneous cell composition and the mixed early-late PD patients in the whole blood cohort, our exon array leukocyte dataset yielded similar proportions of the functional prediction groups to those of the mixed patient’s cohort: 37% and 38% terminal changes and 11% retained introns (compare Figure 2Bii to Figure 2Aii) with more than one functional change following each event.

Functional analysis of the PD AS-modified transcripts using EASE [40] identified the term alternative splicing as having the largest number of assigned genes (i.e. 129) in the advanced patients (p < 0.01 under Table 1, and p < 0.05 under Additional file 4: Table S4), compatible with many of the spliceosome components being subject to self-AS changes [41]. Notably, the advanced cohort presented 1% alternative coding events, considerably less than the 6% observed in mixed patients (as compared with the corresponding matched control volunteers of each tested cohort). In contrast, the fractions of NMD events were similar to the advanced and mixed cohort (5% compared to 4%). Furthermore, the total number of NMD functional predictions was 3-fold larger in the advanced patients (28 compared to 9), possibly reflecting increased NMD effects and decreased variability of blood cell protein composition in purified leukocytes and with disease progression. Thus, NMD events showed an apparent association with the severity of PD.

We have further challenged these observations using the robust FIRMA analysis approach [42]. While in the SI approach, the signal of each exon is normalized by dividing it by the measured gene expression level, the FIRMA approach models the expected behaviour of each exon by calculating its deviation from a robust estimate of the gene expression level. This analysis yielded 167 AS events that occurred in 120 genes overall (Additional file 5: Table S5) in advanced PD patients compared to controls, with 8 of them identified as NMD changes. For example, the vacuolar protein VPS37A gene was detected as both AS- and NMD-changed. Other examples are the phosphodiesterase PDE7A1/3, inhibition of which rescues dopaminergic neurons in cellular and rodent models of PD [43] and the trans-ketolase enzyme TKTL1 [35], which also showed PD association in a gene level analysis of the same cohorts [35]. 29 additional events involved truncation, occurring in non-NMD genes and included five of the 29 PD signature genes that we previously identified in this same cohort: the B cell development controller LRRC8C, the solute carrier protein SLC6A8, the ATPase ATP11B, the cell division cycle protein CDC20 and TKTL1 [35]. Two genes showed both fold-change and modified AS: the tumorigenic keratin KRT7 [44] and the oncogene MED29 [44]. Functional analysis revealed that the FIRMA-detected genes were highly enriched with alternative splicing.

Although there was no overlap between the specific genes identified by the SI and the FIRMA analysis methods, we noted that there was an overlap in terms of gene families between the two lists. For example, the solute carrier (SLC) as well as zinc finger and WD repeat domain-containing genes were detected as significantly changed by both methods. Notably, there was also an overlap between the functional enriched terms of the SI and FIRMA detected genes, for example – alternative splicing, with the SwissProt keyword phosphoprotein as the top detected functional enriched term in both lists, found in 69 of the SI, and 70 of the FIRMA detected genes. The biological process protein transport also appeared as significantly enriched in both lists.

### DBS stimulation-induced AS changes in PD patients leukocytes predicted to yield NMD

Next, we addressed the question of whether the observed splicing changes are correlated with treatment efficacy. We have conduced exon-level SI analysis of blood leukocyte transcripts from PD patients post-DBS, upon symptoms stabilization, and under electrical stimulation (PD-Stim-ON) as compared with one day prior to DBS neurosurgery (patients clinical information is described under [35]). While PD patients showed 319 AS events compared to controls, DBS-induced response of 254 AS changes overall (Figure 2Ci and Additional file 6: Table S6), 44 of which were predicted to induce NMD at the protein level. Thus, although the total number of overall AS events was smaller post- as compared with pre-DBS, the fraction of NMD ones was higher (Additional file 6: Table S6 and Figure 2 C, E), demonstrating considerable DBS-inducible transcript destruction in post-DBS leukocytes. Examples included the erythrocyte membrane protein EPB41L4A with DBS-induced inclusion of an alternative N-terminus, the inflammatory response gene A2M [45], the splicing factor SRRM1 and the I- kB kinase cascade regulator IKBKE. 31 of the identified transcripts were predicted to undergo truncation following the splice changes, of these 9 were also predicted to undergo NMD change. Overall, 80% of the transcripts exhibited more than one splicing event. The functional protein-level implication of the DBS-induced AS changes at the protein level was overall similar to those observed both in advanced and in mixed PD patients pre-DBS as compared with healthy control volunteers (Figure 2Cii, Ciii). The post-DBS NMD predicted transcripts largely differed from the disease-predicted NMD transcripts, with only 3 genes predicted to undergo AS induced NMD changes in both the patients’ leukocytes pre-DBS compared to controls and in the post-DBS stimulated state. Functional sequence and feature enrichment analysis of the detected DBS-modified transcripts revealed enrichment in the following terms: alternative splicing, inflammatory response, metal and zinc ion binding, regulation of cellular response to stress and apoptosis (Table 1 and Additional file 7: Table S7). Functional enrichment analysis of only the NMD predicted transcripts detected alternative splicing, DNA repair and regulation of protein kinase cascade as highly enriched.

Of the DBS-modifiable AS transcripts, 23 were also detected in the PD patients pre-DBS and 2 of those showed DBS-inducible NMD change: the TATA Box gene TAF1D (with inclusion of exon E9-7) and VDAC3, where inclusion of exon E5-1 was predicted to affect functional NMD decay. Four of the DBS-induced AS genes also showed expression change in the same patients: the tumorigenic protein TPRG1, the keratin KRT7, the vesicular trafficking protein EXOC7 and the NMD factor UPF3A, which also changed in patients compared with controls. The treatment-induced AS genes also included interleukin (IL) 19, suggesting immune and NMD relevance to the DBS-induced improvement of motor functions. Although inducing less exon level AS changes as compared with PD, DBS increased the diversity of the detected events to 631 functional predictions (with 40% of the events predictably inducing more than one transcript change, on average). FIRMA analysis of the post-DBS ON- stimulus state as compared with the pre-DBS state detected 110 AS events in 106 transcripts (Additional file 8: Table S8), and 8 of these were also identified by the SI method as subjected to DBS-induced AS changes. FIRMA also identified two genes exhibiting NMD change: the enzyme PCBD2 with post-DBS inclusion of exon 2, and the apoptosis-inducing factor EAF2 that exhibited post-DBS inclusion of exon E10.1.

### The DBS-induced NMD/AS changes are rapidly modifiable and electrical stimulation-dependent

The DBS-induced changes in NMD/AS could potentially be due to the operation procedure, the reduced drug dose and/or the electrical stimulation itself or all of these together. To test these possibilities, we further examined blood leukocytes RNA of the DBS-treated patients following one hour off electrical stimulation (Stim-OFF). We noted 133 AS changes within only one hour following disconnection of the stimulus (Figure 2Di and Additional file 9: Table S9), further decreasing the diversity of the changes to 373 functional predictions (such that every third AS change would predictably modify the protein product of the modified transcript). Functional enrichment analysis once again detected alternative splicing as the top enriched term (Table 1, red color and Additional file 10: Table S10). Of the detected exons, 21 were also identified as undergoing AS changes in the treatment-affected genes (i.e. in DBS stimulation ON-stimulus as compared with the pre-DBS state). Of these, we observed C-terminal PD-related AS changes and DBS-induced NMD changes in the serine protease CORIN, the carbonic anhydrase CA1, the cell division controller TMEM67 and the hematopoietic Krueppel-like factor KLF8. Other detected genes, such as the glycogen phosphorylase PYGM, showed OFF-stimulus dependent expression change.

At the level of functional implications, the fractions of alternative N- and C-termini out of the total functional predictions were comparable with those observed in the ON-stimulus state (36/36% OFF and 37/38%, ON) (Figure 2Dii and Cii, peach and red colors). However, the fractions of truncated transcripts increased OFF stimulation as compared to both pre- and post-ON states (8% as compared with 4% and 5.7%). Also, the fractions of retained introns were similar pre- and post-DBS (11%) but increased upon stimulation cessation (to 13%). In contrast, NMD events declined to 6.2% of the total events as compared with post-DBS on stimulation (Figure 2E), reinforcing the indication that the NMD changes depended on the stimulus. FIRMA comparison of the OFF- and ON-stimulus states, only one hour earlier detected 55 AS events in 55 transcripts (Additional file 11: Table S11). Of the FIRMA detected genes, seven were also detected in the OFF- as compared with the ON-stimulus state through SI analysis, including the fibroblast growth factor receptor FGFR1 [46]. That the DBS-induced capacity to cause NMD/AS changes was largely lost under OFF-stimulus conditions (Figure 2E) further suggested that it was due to the electrical stimulus itself rather than the operation or the modified drug dose.

**Figure 3:**
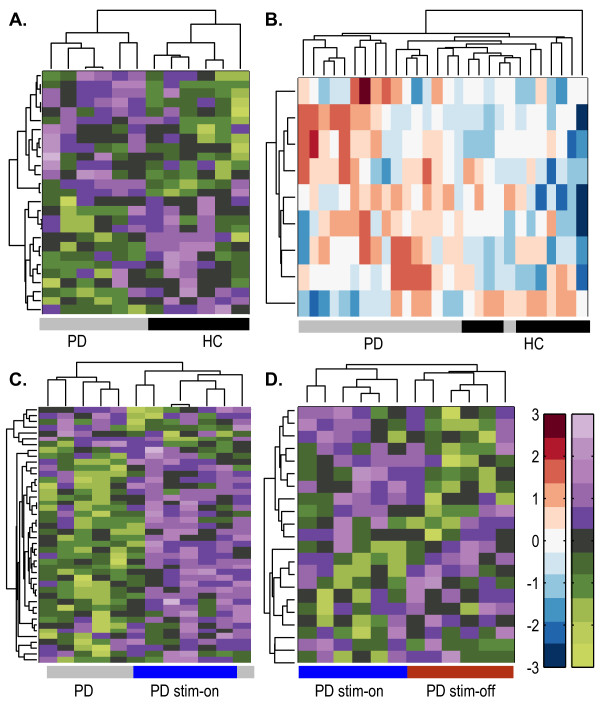
**AS-induced NMD predictions segregate patients from controls and post-treatment states.** (**A**) Hierarchical classification of mixed early-advanced PD whole blood samples by SI values of the changed exon probe sets predicted to induce NMD change. Note that seven of the seventeen patients were mis-classified with the HC group. (**B**) Classification of advanced PD patients’ pre-DBS from matched HC based on the splicing-index (SI) values of those AS exons predicted to induce NMD in blood leukocyte samples. (**C**) HCL classification of patients’ post-DBS ON- stimulus and the same patients pre-DBS correctly classified pre- from post- states based on the SI values of the AS/NMD detections in the Stim-On state. Right: one patient pre-DBS was classified with post-treatment patients. (**D**) HCL classification of PD patients post-DBS following one hour OFF- as compared with ON- stimulus correctly classified all samples through SI analysis of NMD/AS genes by clinical state. Distance measure was correlation in all the classifications and distance measurement method was Euclidian. The color bars represent relative expression ratio (red-higher, blue-lower).

To further challenge the power of the observed differences, we performed hierarchical classification analysis on all of those AS regions that were implicated to cause NMD in the mixed patients’ cohort, and found correct classification of all samples except for 2 misclassifications (Figure 3A). In comparison, directly classifying the expression levels of the regions that exhibited NMD changes correctly distinguished all of our advanced PD patients from controls (Figure 3B). Furthermore, the AS probe-sets predicted to be subjected to NMD under DBS successfully classified the samples ON- stimulus as compared to pre-DBS state based on their expression levels (Figure 3C, PD and PD Stim-On). Finally, the AS probe-sets predicted to be subjected to NMD successfully classified the samples OFF-stimulus from the one hour earlier ON- stimulus state based on their expression levels (Figure 3D, PD Stim-On and PD Stim-Off). Thus, NMD changes emerged as a principal component of the changed disease states of the analyzed patients.

### NMD/AS events alter functional features both with disease progression and under DBS

Post-hoc functional enrichment analysis of the AS genes detected in the mixed published PD cohort revealed enrichment in nucleotide-binding, metal-binding, Natural killer cell mediated cytotoxicity, ATP-binding, transition metal ion binding, ATP binding, gene silencing and positive regulation of immune response (Additional file 2: Table S2). In our advanced PD cohort as compared with age and gender matched healthy control volunteers (HC), post-hoc GO and protein domains enrichment functional analysis [40,47] of the AS modified transcripts likewise detected enrichment for the Molecular Function (MF) term ATP binding and the Biological Processes (BP) terms metal ion transport, in addition to mitochondrion organization (Table 1 and Additional file 2: Table S2), both of which also emerged as PD-relevant in brain transcriptomes from mouse PD models [48]. The AS-modified genes further included the PD-related ubiquitin ligase component FBXO9 [49] which exhibited inclusion of an N-terminal exon in PD leukocytes. Functional analysis of only AS transcripts predicted to cause NMD functional change detected significant enrichment in the cell cycle checkpoint GO term (Table 1).

The genes that underwent AS change following DBS revealed the highest enrichment in splice variant sequence feature (which appeared in 28 of the NMD predicted genes), the Swissprot database term alternative splicing (Table 1 and Additional file 7: Table S7), and the GO BP terms alternative splicing, oxidative phosphorylation, mitosis, protein transport, leukocyte proliferation and ATP-binding. Those genes that presented AS-induced NMD functional prediction following DBS exhibited enrichment in regulation of protein kinase cascade, cellular response to stress, alternative splicing, DNA damage, DNA repair and disease mutation (Table 1, lower panel). Highlighting the role of NMD in the DBS-inducible leukocyte AS changes, the GO BP term nuclear-transcribed mRNA catabolic process, nonsense-mediated (NMD) decay was also enriched in the group of treatment-increased AS events.

Comparing post-DBS leukocyte exon expression of the OFF- to the one hour earlier ON-Stimulation state highlighted 63 and 61 genes of the alternative splicing and sequence feature splice variant terms, with metal ion-binding site: Magnesium 2 as the top sequence feature detected. Additional enriched pathways included complement alternate pathway activation (hinting at immune involvement under OFF- state), acute inflammatory response and mitochondrion (Additional file 10: Table S10). Narrowing of the functional enrichment analysis to only the NMD fraction of the detected AS transcripts detected enrichment in alternative splicing, highlighting the rapid, NMD-linked, molecular signature reversal of stimulation treatment following the one hour OFF stimulus. Thus, while the different interrogated states showed variable enrichment features, all shared AS-related elements as primarily affected.

## Discussion

Despite its biological importance, the functional and dynamic implications of NMD changes as well as the link between splicing and functional NMD implications were never tested in human patients. Overall, an inclusion or exclusion of a single probe set often reflect more than one functional change; and those events that modified N- and C- termini were frequently accompanied in our datasets by functional NMD prediction. Specifically, in PD patients’ blood cells and leukocytes pre- and post-DBS, ON- and OFF- stimulus, we identified AS changes that predictably induce NMD, modify N- and C- termini, retain introns, alter coding events (defined as a change in internal, rather than terminal protein sequence) and cause truncation (a splicing event that causes a reduction of at least 50% in the protein product size (Figure 1), or induce various combinations of those changes. We detected more events in advanced PD patients’ leukocytes than in whole blood cells from the mixed independent cohort, with further increases following DBS stimulation and rapid decreases under OFF-stimulus. The fraction of leukocyte NMD events increased from 5.3% pre-DBS to 7% post-DBS and declined again upon stimulation cessation to 6.2%. Moreover, the number of NMD causing events was higher post-DBS as compared with both pre- and post- OFF states as well as the mixed cohort of early-late PD, and the observed AS changes OFF- stimulus that were accompanied by returning tremor symptoms also wiped off much of the DBS-mediated AS changes in leukocyte mRNA. A comparison of purified leukocyte RNA from advanced patients to the whole blood preparation from the published mixed cohort revealed that both the splicing and NMD implications were massive in leukocytes from advanced patients as compared with mixed early and advanced whole blood cells. Together, these findings reflect progressive NMD increases with disease progression and treatment-induced changes, dependent on the electrical stimulus; and suggest that the NMD process is one of those checks and balances mechanisms regulating RNA metabolism under modified brain-to-body signalling.

One common outcome of AS events is suppressed function of a gene by the production of non-functional transcripts, which can be achieved through the NMD mechanism [50-53]. NMD was first identified in the late 1970’s in thalassemic carriers of nonsense globin mutations as the cause for decreased abundance of affected mRNA transcripts, rather than production of truncated proteins [54]. Recently it was discovered that NMD also regulates normal gene expression [55], together suggesting that NMD manipulations could potentially cause both loss-and gain- of -function changes and be of clinical benefit. AS/NMD coupling was suggested before [56] and was termed ‘regulated unproductive splicing and translation’ (RUST) [11,50,57]. Correspondingly, the identified NMD/AS changes reflect DBS-inducible enhancement of cellular response to stress and alternative splicing, both were rapidly modified by stimulation cessation to NMD-induced functional changes in immune and inflammation properties. We observed DBS-stimulus NMD predictions in genes participating in DNA damage and repair and alternative splicing, compatible with our findings of bidirectional risk-protection role of these pathways by meta-analysis of mouse PD model brain transcriptomes [48]. Overall, this study expands our observations for disease-related AS changes in both brain and blood cells. To the best of our knowledge, this is the first demonstration of splicing-mediated NMD irregularities under disease and of their correction through DBS-related brain-to-body messages, changing transcript profiling in blood leukocytes as a symptoms-related mechanism of action.

PD prevalence (0.5-1% from 65–69 years, 1-3% over 80 years [58] and 4-5% over 85 [20]) is predicted to grow worldwide within the next decades [59], highlighting the importance of developing new therapeutic strategies which should ideally target the disease-related impairments. In this context, 50% of known disease-associated mutations in RNA-binding proteins cause nervous system diseases [60,61]. Examples include SMN2 mutations, which induce motor neuron degeneration in spinal muscular atrophy [62,63], and mutations in the RNA-binding proteins TDP-43 and FUS, which induce amyotrophic lateral sclerosis [64-66]. Also, neuron-specific splicing factors such as NOVA regulate transcript categories that are essential for neuronal functioning [67], and transcriptome profiling demonstrates altered brain AS events in various engineered mouse models of human degenerative diseases, PD among them [63,68,69]. Thus, global changes in AS regulation may contribute to the initiation and/or progression of neurodegenerative processes. Therefore, searching for PD-related mechanism(s) related to RNA metabolism is called for. In this context, NMD has a role in preventing dominant diseases and can also eliminate mRNAs that would otherwise result in the production of partly or fully functional truncated proteins. In such instances, interventions to prevent degradation of transcripts containing PTCs may be therapeutically useful. Approaches to protect mRNAs containing a PTC from NMD, thereby promoting the synthesis of a functional protein, have been explored for cystic fibrosis, Duchenne muscular dystrophy, Hurler syndrome and X-linked nephrogenic diabetes insipidus [3]. Modulation of either the PTC of the disease-associated AS transcripts, or of those genes regulating the NMD process, through RNA interference, may enable the development of NMD-targeted therapeutics in the future.

DBS is increasingly used for other human diseases including depression [70] and chronic pain [71] where it is predicted to serve for treating tens of thousands of people in the world during the next decade. In addition to its established use for treating the deliberating motor symptoms of PD [72], DBS has also been proven to be a powerful tool for alleviating the symptoms of the treatment-resistant motor diseases essential tremor [73] and dystonia [74]. Additionally, DBS of a variety of brain regions is being increasingly tested to treat a growing number of non-motor diseases including behavioural ones. These include obsessive-compulsive disorder (OCD), major depression [75] and Tourette syndrome [76]. However, the mechanism of action of DBS therapeutics is still obscure. Our current study opens new venues to exploring the molecular mechanisms by which DBS affects motor and cognitive symptoms of both PD and other neurological disorders.

## Conclusions

**Figure 4:**
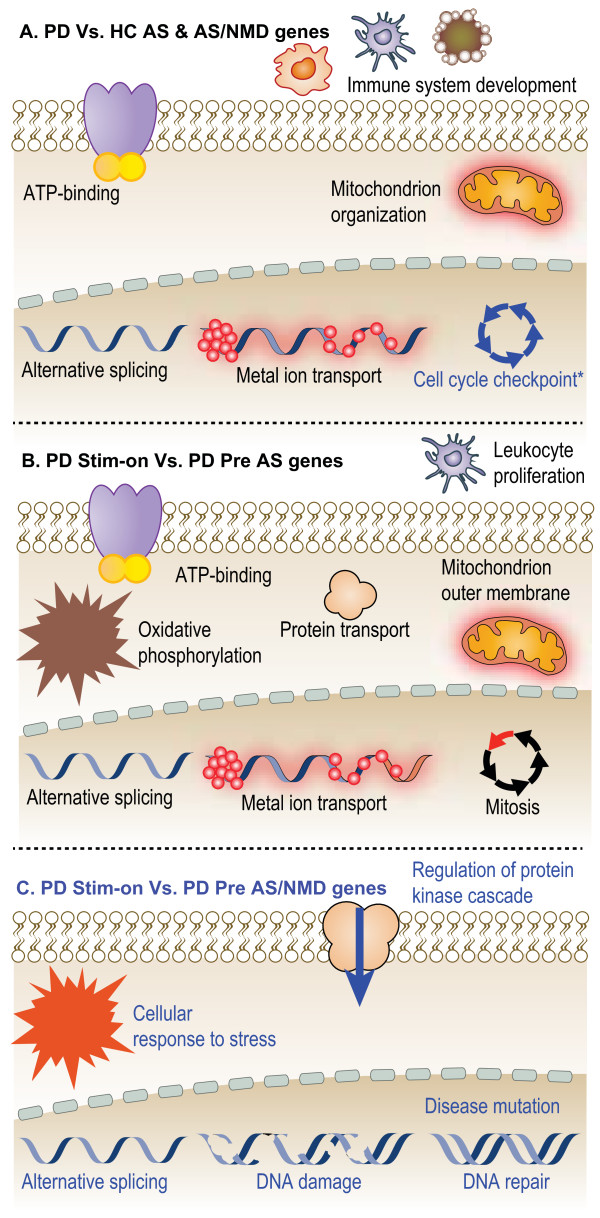
**Post-DBS NMD spliced transcripts correct disease pathways.** (**A**) PD patients’ pre-DBS exhibited functional changes of AS genes enriched in *immune system development*, *ATP-binding*, *mitochondrion organization*, *alternative splicing* and *metal ion transport.* NMD/AS genes were enriched in *cell cycle checkpoint process*. (**B**) DBS treatment induced functional changes in *leukocyte proliferation, ATP-binding*, *Oxidative phosphorylation*, *protein transport*, *mitochondrion outer membrane*, *alternative splicing*, *metal ion transport* and *mitosis*. (**C**) DBS-induced NMD/AS functional enrichment included *regulation of protein kinase cascade*, *cellular response to stress*, *alternative splicing*, *DNA damage*, *DNA repair* and *disease mutation*.

We identified dynamic changes of AS-induced NMD assessed events in PD patients’ blood cells and following deep brain electrical stimulation. Specifically, we noted that both the disease and yet more so, the DBS treatment induced NMD prediction increase with disease-relevant functional implications as compared with both healthy control volunteers and the pre-surgery state. Intriguingly, those NMD events that were identified in the mixed cohort of early and advanced patients were reminiscent of those in the advanced cases, albeit less discriminating. A surprisingly rapid decline in both AS and NMD events occurred following an hour OFF-stimulus, and Alternative Splicing changes emerged as a commonly modified function in all tested stages, with known PD-related pathways identified as enriched in AS-induced NMD changes (Figure 4). Zooming into NMD events showed classification between advanced PD patients and controls, an inverse pattern of changed NMD events following DBS and reversal of that pattern in OFF-stimulus samples. DBS is rapidly emerging as a promising therapy for various neuronal disorders and is increasingly applied for treating movement disorders (PD, dystonia and essential tremor), and increasingly tested to treat other diseases such as epilepsy and major depression. DBS may become in the future tailored and patient-specific, utilizing specific target regions for individual clinical manifestations. The findings of this study may therefore have future implications for exploring and interfering with the impaired molecular mechanisms that underlie not only neurodegenerative and neurological disorders but also other DBS-treatable conditions. In particular, our findings are relevant for searching for novel PD-targeted therapeutics.

## Methods

This study was authorized and approved by the Ethics committee of the human review board (Hadassah University Hospital, Ein-Kerem, approval number 6–07.09.07) in accordance with the Declaration of Helsinki principles. Following oral agreement, all participants signed informed consent prior to inclusion. The decisions regarding the DBS treatment were taken solely by physicians at the Hadassah Ein Kerem Hospital and the PD patient volunteers were only subsequently recruited for the study.

### Independent PD whole blood microarray cohort

28 exon array raw CEL files of whole blood mRNA from 17 mixed early and late PD patients (time from diagnosis: 2–23 years) and 11 healthy control volunteers (HC) [34] were obtained from the Gene Expression Omnibus (GEO) database.

### Patient and controls recruitment, DBS neurosurgery and clinical evaluation

Seven PD male patients nominated for bilateral DBS neurosurgery were recruited to the study and six healthy age-matched male controls (HC). Subjects were assessed for their clinical background and state and fulfilled detailed medical history questionnaires. Patients with other medical conditions were excluded, including depression and past and current DSM Axis I and II psychological disorders (SM), chronic inflammatory diseases, coagulation irregularities, previous malignancies or cardiac events, or any other surgical procedure up to 1 year pre-DBS. Exceptions were hyperlipidemia (present in two patients and three control volunteers), hypertension and diabetes (two patients each). All patients went through bilateral STN-DBS electrode implantation (Medtronics, USA) and were under dopamine replacement therapy (DRT) both pre- and post- DBS, with a significantly reduced dosage post-DBS (t-test p < 0.01). The last medication was administrated at least 5 hours pre-sampling. The patients and healthy volunteers also exhibited similar total white and red blood cell counts. Further clinical details (such as age, disease duration and DRT dosage) are given under the supporting information of [77]. Blood samples were collected from each patient at three time points: (1) one day pre-DBS upon hospitalization, with medication (2) post-DBS ON-stimulus (range 6 – 18 weeks) (Stim-ON), when reaching optimal clinical state as evaluated by a neurologist and under a lower therapy dose, and (3) OFF-stimulus, following 60 minutes OFF electrical stimulation (counted from stage 2) (Stim-OFF). Healthy control volunteer samples were collected from six age- and gender-matched academic staff researchers at the Edmond Safra campus of the Hebrew University of Jerusalem. Blood sample collection, RNA extraction and microarray sample preparation, hybridization and scanning were all as detailed [27].

### Probe-set summation

Microarray probe-set summation of the input CEL files was conducted using Affymetrix Power Tools (APT) through AltAnalyze.

### Exon-level alternative splicing analysis

Splicing targeted analysis of both sets of exon array data was conducted using the software AltAnalyze using the Ensembl human database (#37) (http://www.ensembl.org). Briefly, AS targeted analysis was conducted using both Splicing-Index (SI) [78,79] and Finding Isoforms using Robust Multichip Analysis (RMA) linear model fitting [42]. Prior to the analysis, probe-sets with detection above background (DABG) p-value were removed from the input data. Overall, following the filtering, 487,571 core probe sets remained in our in house data set for analysis and 238,749 in the independent data set. Expression data was formatted to log scale. The SI is derived by subtracting the group-normalized intensity of the patient group pre-DBS from control samples or from post-ON state or post-ON from post-OFF states. The SI represents the ratio of the exon intensities between the two tested conditions following normalization to the gene intensities and serves to measure the exon inclusion level. The SI value is calculated as follows: the gene-level normalized probe-set intensity is defined as $\mathrm{NI}=\frac{\mathrm{probeset}\;\mathrm{intensity}}{\mathrm{gene}\_j\;\mathrm{level}\;\mathrm{intensity}}$ for the j-th gene and the splicing-index value subsequently calculated as $\mathrm{SI}=\log2\;\left(\frac{\mathrm{NIps}\_i}{\mathrm{NIps}\_i}\right)$ ps_i: the i-th probe set intensity of the j-th gene. All calculated SI values were normalized using the NI equation to the constitutive gene expression level. AS change was detected using one-way ANOVA (equivalent to t-test) in order to probe for differential inclusion of exons into genes. In the case of post- compared to pre-DBS and OFF compared to ON stimulation analyses, a paired t-test was conducted. The significance of microarray pair-wise comparisons was derived by 2-tailed t-test on the SI calculated values. To detect the highest proportion of true positives the analyses included only the following: (1) probe sets with Detection Above Background (DABG) p-value > 0.05 (2) exons with a gene level normalized log ratio between experimental conditions > 2, (3) t-test p-values < 0.05 (4) FDR p-value < 0.05 (5) maximal absolute gene expression change = 3 and (6) core level probe sets. The Ensemble database served, through AltAnalyze, to map each alternative splicing event to the corresponding functional prediction (e.g. NMD) either directly or through comparison of all transcripts for that gene.

### Pathway analysis

Pathway post-hoc analysis on the detected AS transcripts was conducted through the DAVID [80,81] EASE [40] functional enrichment analysis module. The identified biological themes included Gene Ontology (GO) [82] terms, KEGG pathways [83], Swissprot (SP) [84] sequence features and Protein Information Resource keywords (SP-PIR), the InterPro protein families database [85] and the BioCarta DataBase (http://www.biocarta.com/).

### Availability of supporting data

The data-sets supporting the results of this article are available in the GEO repository, http://www.ncbi.nlm.nih.gov/geo/query/acc.cgi?acc=GSE23676 and http://www.ncbi.nlm.nih.gov/geo/query/acc.cgi?acc=GSE18838.

## Abbreviations

AS: Alternative plicing; BP: Biological process; DBS: Deep brain stimulation; GO: Gene Ontology; GEO: Gene Expression Omnibus; MF: Molecular Function; NMD: Nonsense-Mediated decay; FIRMA: Finding Isoforms using Robust Multichip Analysis; PD: Parkinson’s disease; PTC: Pre-termination codon; SI: Splicing Index; UTR: Un-Translated Region.

## Competing interests

The authors declare that they have no competing interests.

## Authors’ contributions

LS, HB, ZI and HS designed the study, HB, ZI and LS submitted for Helsinki ethics committee approval and recruited patients and healthy control volunteers to the study, LS signed the participants of agreement forms, produced the blood leukocyte samples and RNA and conducted the bioinformatic data analyses. LS drafted the manuscript with the help of HS and all the co-authors approved the final manuscript. All authors read and approved the final manuscript.

## Supplementary Material

Additional file 1: Table S1 Alternatively spliced genes detected by splicing-index exon level analysis of the independent mixed early-late PD patients and healthy control volunteers (HC) cohort. Click here for file

Additional file 2: Table S2 Functional terms, functions and processes found as enriched in the detected external cohort alternatively spliced genes. Click here for file

Additional file 3: Table S3 Alternatively spliced genes detected by splicing-index exon level analysis of the in-house advanced PD patients and HC cohort. Click here for file

Additional file 4: Table S4 Functional terms, functions and processes found as enriched in the in-house PD cohort alternatively spliced genes detected upon comparison to HC. Click here for file

Additional file 5: Table S5 Alternatively spliced genes detected by robust isoform finding analysis (FIRMA) of the exon microarray data of the in-house advanced PD patients and HC. Click here for file

Additional file 6: Table S6 Alternatively spliced genes detected by splicing-index (SI) exon level analysis of the in-house advanced PD patients cohort post-DBS as compared with pre-DBS (pre-treatment) state. Click here for file

Additional file 7: Table S7 Functional terms, functions and processes found as enriched in the in-house PD cohort alternatively spliced genes detected upon comparison of pre- to post-DBS (treatment) states. Click here for file

Additional file 8: Table S8 Alternatively spliced genes detected by FIRMA analysis of the exon microarray data of the in-house advanced PD patients cohort post-DBS as compared with pre-DBS (pre-treatment) state. Click here for file

Additional file 9: Table S9 Alternatively spliced genes detected by splicing-index exon level analysis of the in-house advanced PD patients cohort post-DBS ON-Stim as compared to one hour off stimulation state (following 1 hour of cessation of the electrical stimulator: OFF-Stim). Click here for file

Additional file 10: Table S10 Functional terms, functions and processes found as enriched in the in-house PD cohort alternatively spliced genes detected upon splicing-index analysis of the two tested post-DBS states (on and off electrical stimulation:ON-Stim and OFF-Stim). Click here for file

Additional file 11: Table S11 Alternatively spliced genes detected by robust isoform finding analysis (FIRMA) of the exon microarray data of the in-house advanced PD patients in the two post-DBS states (on and off electrical stimulation:ON-Stim and OFF-Stim). Click here for file
